# Effect of Sport Activity on Uncomplicated Bicuspid Aortic Valve: Long-Term Longitudinal Echocardiographic Study

**DOI:** 10.3390/jcdd11090285

**Published:** 2024-09-10

**Authors:** Massimiliano Bianco, Fabrizio Sollazzo, Gloria Modica, Isabella Carlotta Zovatto, Rachele Di Mario, Riccardo Monti, Michela Cammarano, Vincenzo Palmieri, Paolo Zeppilli

**Affiliations:** 1 Unità Operativa Complessa di Medicina dello Sport e Rieducazione Funzionale, Fondazione Policlinico Universitario Agostino Gemelli IRCCS, Università Cattolica del Sacro Cuore, 00168 Rome, Italy; massimiliano.bianco@policlinicogemelli.it (M.B.); fabrizio.sollazzo@unicatt.it (F.S.); isabella.zovatto@gmail.com (I.C.Z.); rachele.dimario25@gmail.com (R.D.M.); riccardo.monti@guest.policlinicogemelli.it (R.M.); michela.cammarano01@gmail.com (M.C.); vincenzo.palmieri@unicatt.it (V.P.); paolo.zeppilli@unicatt.it (P.Z.); 2 Dipartimento di Neuroscienze, Università Cattolica del Sacro Cuore, Largo Agostino Gemelli 8, 00168 Rome, Italy

**Keywords:** sport, athlete, bicuspid aortic valve, echocardiography, follow-up, pre-participation screening

## Abstract

Background: The bicuspid aortic valve (BAV) is a congenital heart defect that can lead to certain complications (aortic stenosis, regurgitation, dilatation and endocarditis), the diagnosis and clinical monitoring of which are effectively entrusted to transthoracic echocardiography (TTE). The impact of training on the natural history of the disease remains unclear. Methods: A retrospective cohort of athletes with uncomplicated BAV aged 18–50 years, who underwent at least 2 TTEs with a minimum follow-up of 5 years, subdivided according to the level of physical activity during follow-up into ‘’untrained’’ and ‘’trained’’, was collected. RESULTS: 47 athletes (87.3% male, median 21.0, (18.0; 33.0) years) were included. Median follow-up was 11.6 (8.4; 16.3) years. No statistically significant difference in the growing rate of aorta, left ventricle, nor a significant worsening of aortic stenosis and regurgitation was found. Moreover, there was no significant correlation between weekly training minutes during follow-up and the echocardiographic parameters related to heart size and function. Conclusions: In BAV without major complications, high training volumes do not correspond to a more rapid and significant deterioration in valve function nor to a more rapid increase in aortic or cardiac chamber size.

## 1. Background

Bicuspid aortic valve (BAV) is one of the most common congenital heart disease, occurring in about 0.5–2.5% [1,2] of the population, with a male to female ratio of 3:1 [1]. Instead of the usual three cusps, the two cusps of a bicuspid valve are typically larger and thicker. Based on the type of valve opening, it is possible to delineate a BAV with the aortic valve leaflets arranged in a “latero-lateral” position, and a more prevalent form of BAV with the aortic valve leaflets arranged in an “antero-posterior” position, which demonstrate different behavior over time in developing complications [3].

Most individuals with a BAV lead normal lives without experiencing any significant symptoms or complications. However, some individuals with this condition may develop certain complications over time, including aortic stenosis [3], aortic regurgitation [3] and aortic aneurysm [3].

Treatment for BAV depends on the severity of symptoms and complications. Mild cases may only require periodic monitoring, while more severe cases may necessitate medication or surgical intervention, such as valve repair or replacement [4].

BAV may be present in isolated form or in association with other congenital heart diseases, such as aortic coarctation [3], interventricular defect [4], the hypoplastic left ventricle [5] and syndromes characterized by aortic dilatation (Marfan syndrome, Loeys–Dietz syndrome [6]) or obstruction at the left ventricular outflow tract (Turner syndrome, Shone syndrome, Williams syndrome [7]) and coronary anomalies [5].

The diagnosis is made on two-dimensional echocardiogram, with the finding of two cusps instead of three in the short axis projection in systole and diastole [4]. The clinical relevance of the condition, with the spectrum of its possible complications, becomes even more relevant in the context of sports practice, where the impact of regular training on the natural history of the disease has not yet been fully elucidated [2,8,9].

Predominantly static or isometric exercises result in work characterized by increased peripheral resistance and marked increase in arterial pressure (“pressure” work), which in the long run could be a risk factor in the context of BAV, and by constitution more sensitive to parietal stress and more prone to dilatation [10]. 

For this reason, over time, the competitive sports guidelines dealing with sports fitness in athletes with BAV have always taken a cautious attitude toward those athletes who showed signs of progressive deterioration of valve function, and progressive dilatation of the aortic root or left ventricle [6,7,11,12]. Moreover, the impact of BAV on arrhythmic risk still has room for further investigation [13]. In any case, there is a very meager body of studies that have been concerned with assessing the natural evolution of BAV in athletes [7,11], most of which present short-term [12,14] follow-up. In this sense, long-term data on the echocardiographic course of BAV according to the volume of exercise practiced could be of significant added value in identifying levels of physical activity associated with an increased occurrence of long-term complications. The primary aim of the study is to outline the long-term BAV evolution profiles based on the level of cardiovascular commitment to which the subject is subjected, and possibly to identify the presence of a correlation between athletic workload and BAV evolution.

## 2. Materials and Methods

### 2.1. Study Population

We built a retrospective cohort of the case history of athletes who were either seen at our Sport Medicine Ambulatory for first level investigations related to pre-participation screening, following which a diagnosis of BAV was made, or of athletes who came to our practice for a second-level evaluation following a diagnosis of BAV already made elsewhere. All these athletes were assessed at least twice by echocardiography, at least five years apart. These athletes were subdivided based on the level of sporting activity practiced during the period between the first visit and the follow-up: in fact, one group of athletes had, by their own choice, discontinued or markedly reduced the level of sporting activity practiced, while a second group continued to practice sport with a volume of work not dissimilar to that previously practiced. In particular, the level of physical activity was measured as objectively as possible at follow-up by administering the International Physical Activity Questionnaire (IPAQ), a commonly used questionnaire for assessing the level of daily physical activity, as it is validated and reproducible [15]. In this regard, as per the questionnaire, subjects practicing at least 2520 METs per week were considered “trained”, while all those who remained below this level were considered ‘untrained’. By way of example, practicing at least 45 min per day of highly cardiovascular demanding sporting activities (e.g., individual or team sports at a competitive level, except for juggling sports), or 90 min per day of moderately cardiovascular demanding activities (e.g., slow cycling, gym activities, gardening) entitled the subject to be classified as “trained”.

On the clinical-anamnestic level, in addition to the training load information collected by means of the IPAQ, information was collected concerning family history of cardiovascular disease, cardiovascular symptoms and the presence of other known concomitant heart diseases; nevertheless, information was collected concerning the initiation of cardioactive therapy (in particular, in relation to drugs such as anti-hypertensives that may influence the natural course of BAV), and concerning the occurrence of major cardiac events (major arrhythmias, aortic aneurysms and dissections, heart failure, stenosis and/or more than moderate degrees of insufficiency) and the possible need for surgical correction of BAV complications was noted.

The anamnestic collection was supplemented by a general objective examination, including anthropometric assessment (weight, height, Body Mass Index (BMI) and Body Surface Area (BSA)).

In all patients, a resting electrocardiogram (ECG) was acquired, from which any abnormalities present were detected (AV and IV conduction abnormalities; Q waves; reduced QRS voltages; ST-tract or T-wave abnormalities; presence of brady and/or tachyarrhythmias).

In relation to the echocardiogram, general (intracavitary dimensions, biventricular systolic function, global and segmental kinetics abnormalities, valve morphology and function, diastolic function) and BAV-specific parameters (BAV morphology classified according to the most recent international consensus [16]), aortic root dimensions, presence of aortic valve stenosis and/or insufficiency and its grading) were collected, again both at initial and remote follow-up.

### 2.2. Inclusion and Exclusion Criteria

All athletes of both genders with an established diagnosis of BAV, who at the time of the initial assessment were practicing sport at a recreational, competitive or professional level (classified as “trained” according to IPAQ) were included in the study. The individuals included had to be between 18 and 50 years old at the time of the first echocardiographic evaluation and had to have been re-evaluated for a period of at least 5 years by transthoracic echocardiography (TTE).

All those who presented additional cardiovascular diseases (including arterial hypertension), evidence of major complications associated with BAV at the time of initial diagnosis (i.e., the finding of aortic dilatation, endocarditis and/or the presence of more than mild stenosis and/or insufficiency) and those for whom it was not possible to ascertain complete information on the degree of training and the echocardiographic parameters of interest, both at initial assessment and at follow-up, were excluded from the present study.

Finally, as regards those who had undertaken cardioactive drug therapy and/or undergone corrective cardiac surgery related to BAV, they were included in the study for the follow-up period prior to the occurrence of these events.

### 2.3. Echocardiographic Assessment

Transthoracic echocardiography was performed with the Toshiba Artida SSH-880CV (Canon Medical System Corporation, Ōtawara, Tochigi, Japan). General information on biventricular size and function and the presence of congenital and/or acquired heart disease was collected, thus including linear dimensions and parietal thicknesses of the left ventricle; systolic and diastolic function of the left ventricle; linear dimensions of the right ventricle; linear dimensions of the left atrium; morphology and function of the pulmonary, mitral and tricuspid valves; presence of signs of interatrial or interventricular shunt; presence of regional parietal kinetics abnormalities; and presence and degree of pericardial effusion, if any.

In relation to BAV, the following data were collected: morphology of the BAV, distinguished into 2 forms (“antero-posterior” and “latero-lateral”), assessment of valve function (stenosis, insufficiency and relative grading), evaluation of aortic root size in parasternal long axis projection at 4 points (aortic annulus, sinuses of Valsalva, sino-tubular junction, ascending aorta at least 1 cm above the sino-tubular junction). Measurements were taken using leading edge to leading edge methodology in M-mode, at the end diastole (apart the left atrium, that was measured at end systole) and the aortic point of maximum dilatation was sought for each athlete, looking for maximum perpendicularity to the long axis of the aorta. Every single degree of deterioration of valvular insufficiency and/or stenosis (including the transition from trivial to mild) was considered as worsening of valvular function.

## 3. Results

**Figure 1:**
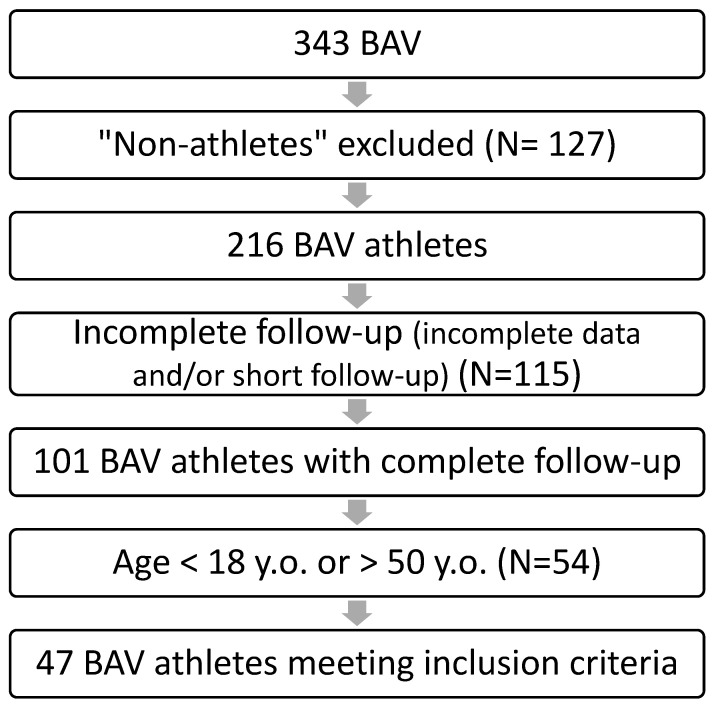
Applying exclusion criteria on our BAV population, initially assessed for competitive eligibility or for second-level assessments related to BAV.

Our population consisted of 343 BAV, but only 47 athletes fitted by our strict inclusion criteria (Figure 1): they were mostly male (87.2%) with a median age 21.0 (18.0; 33.0) years (Figure 1). The duration of follow-up was 11.6 (8.4; 16.3) years: during this time, there were no statistically significant variation in the main anthropometric parameters of the population (*p* > 0.05), except for the weight, which increased in the whole population between the two examinations (Table 1). 

**Figure 2:**
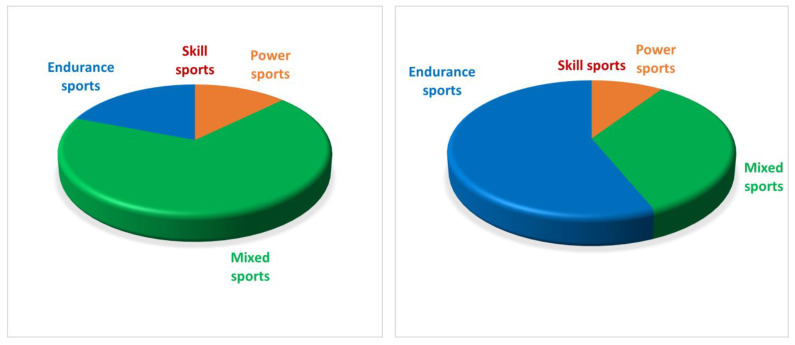
Differences in sports practised and cardiovascular commitment according to the European Society of Cardiology classification at first evaluation (**left**) and follow-up (**right**).

This population at the initial time consisted of 21 (44.7%) non-competitive athletes and 26 (55.3%) competitive or professional athletes: both the sports practised and their cardiovascular commitment, derived from the European Society of Cardiology guidelines, showed substantial differences between initial assessment and follow-up [17] (Figure 2).

For this reason, we decided to objectively classify the training volume by deducing it from the IPAQ questionnaire commonly administered to our athletes: in this way, it was possible to divide the population into a group of athletes who, by personal choice unrelated to aortic valve pathology, have stopped regular training and can therefore be considered “untrained” (19, 40.4%) at the follow-up (UFU), and a group of athletes who continued with a similar training volume during the period between the initial assessment and the follow-up, who can therefore be classified as “trained” (28, 59.6%) at follow-up (TFU). 

**Figure 3:**
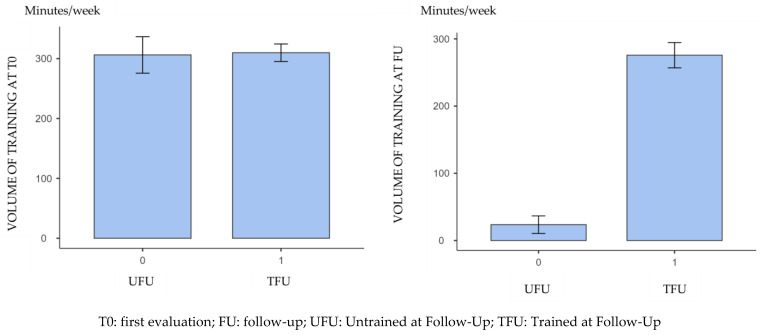
Comparison of weekly training volume (in minutes per week) between participants who declared to have stopped regular training during the follow-up and participants who declared to have continued regular training, according to IPAQ results.

Through this subdivision, it is indeed possible to highlight the different behaviour of the training load between initial assessment and follow-up (Figure 3), whereas in the group of ‘trained’ athletes there was no statistically significant difference in the training minutes performed between initial assessment and follow-up (*p* = 0.129).

As for BAV morphology classification, we found 33 people (70.3%) with “antero-posterior” morphology and 14 people (29.7%) with “latero-lateral” morphology. In the whole population, the comparison of the morpho-functional parameters over time showed a statistically significant change for all (*p* < 0.05). Even evaluating the two groups (trained and untrained) separately regarding morpho-functional parameters, most of the parameters showed a statistically significant increase in the time between the two evaluations (Table 2). However, we did not consider this to be sufficient, as we were primarily interested in verifying whether a constant training load led to a faster rate of growth of the aorta size.

For this reason, we calculated the rate of variation for each echocardiographic parameter between the first visit and follow-up, as follows:

X_var_ = (X_FU_ − X_T0_)/X_T0_ × 100, (1)

where

X_FU_ = generic parameter at follow-up;
X_T0_ = generic parameter at first evaluation

This analysis made it clear that there was not a different growth rate of the aortic dimensions in the two groups; moreover, there were not any substantial changes in the main cardiac morpho-functional parameters between the population of trained and untrained people throughout the study duration (*p* > 0.05 for all) (Table 3).

## 4. Discussion

The high prevalence of BAV in the general population places the sports physician in charge of deciding on the competitive clearance of athletes with this pathology with the persistent doubt of whether the practice of sport may be detrimental in the long term both to valvular function and to the size of the aorta and cardiac chambers, since the high cardiovascular effort, by increasing systolic output, heart rate and blood pressure significantly, could act as a negative stimulus in the context of BAV, in which the abnormal valvulo-genesis is probably linked to a general anomaly of the extracellular matrix at molecular level [18].

In particular, the increased size of the aortic root is a major concern because of the risk of rupture that aneurysmal development at the aortic bulb and ascending root brings with it.

Our data are additive but complementary to some of the conclusions that the literature had already reached on the subject: Boraita et al. [11] in a case-control study on a group of elite Spanish athletes with BAV had already concluded that there was no difference in aortic size and function between them and a group of untrained people with the same pathology. The same study had also carried out a short follow-up on a small subgroup of athletes, showing no worsening of the parameters analyzed over time, except for the proximal section of the ascending aorta.

The prospective study of Spataro et al. [19] also highlighted how the continuation of sporting activity was not in itself responsible for the worsening of BAV, although it did note that some subjects initially with uncomplicated BAV had shown a clinical worsening with the appearance of symptoms without any specific causal factor being highlighted.

Finally, similarly, the work of Stefani et al. [2] showed that the presence of aortic bicuspid with no more than mild valvular dysfunction was not an additional risk factor for the development of dysfunction and increased left ventricular size in a group of athletes.

Our study reaches similar conclusions by analysing a prolonged follow-up on a cohort of athletes of various sports but practising rather high workloads, in which the only essential discriminating element is represented, in one of the two ‘arms’ of the cohort, by the interruption of significant volumes of physical activity. In this sense, we consider the data provided by our work to be very relevant, because they support the assertion that even high volumes of physical activity in the context of a BAV not affected by major complications do not represent a clear worsening factor for cardiac and valvular morphology and function. This aspect makes it possible to observe such a valvular abnormality with more tranquillity and less suspicion when selecting the athletic training load.

Considering the specific effect that power sports activity has on the size of the aortic root [20], the natural development of our study should consider the comparison over time of the evolution of the BAV between athletes trained for power sports, athletes trained for mixed or endurance sports and untrained persons: such a study, particularly if structured prospectively, would provide essential information in defining even better the risk profile of the athlete who undergoes high volumes of training despite being the bearer of a BAV.

In any case, on the basis of the data at our disposal to date, it remains a common-sense suggestion to closely monitor the echocardiography of all athletes suffering from BAV, in order to highlight any deterioration of the clinical-instrumental picture at an early stage and to be able to take the most appropriate countermeasures. Based on our clinical long-time experience, we suggest a heart ultrasound check yearly in athletes with “almost normal” BAVs, whilst we advise a closer follow-up (6 months) in athletes with moderate aortic gradient, insufficiency and/or ascending aortic dilation. In the years to come, an increasingly significant amount of data within the athlete’s BAV would make it possible to identify the specific factors that, even in the presence of “almost normal” BAV, are associated with a progressive cardiovascular deterioration, so as to better define the criteria for granting sports eligibility.

In analysing the results of our work, we cannot overlook some important limitations, including firstly the modest sample size and the small female component of the sample, which do not allow for full generalisability of the results.

Furthermore, as we did not consider any athletes who had already developed signs of major complications (moderate stenosis or insufficiency, dilatation of the aortic root or ascending aorta), it was not possible to express an opinion on the effect of sports practice in these specific situations.

Finally, if the classification of physical activity by means of IPAQ allows us to have an idea of the load of daily physical effort sustained by the participants in the study, it is not sufficient to have a clear idea of the functional capacity of the athletes themselves, and is therefore not ideal for sustaining a correlation between exercise capacity and the clinical progression of the pathology, nor, as anticipated, does it allow us to understand whether certain specific modes of exercise (e.g., power sports, team sports, massive aerobic–anaerobic sports) are more correlated with a greater degree of clinical deterioration.

## 5. Conclusions

Our study shows that two populations with BAV objectively distinguished by level of physical activity practised (measured by IPAQ questionnaire) do not show a statistically significant variation in either the main morphological parameters of the aortic root and left ventricle, nor a different progression of aortic valve function. No correlation was shown between the minutes of weekly physical activity practised and the morpho-structural changes of the BAV.

Therefore, based on our data, we can assert that, in the context of BAV without major complications (“near-normal” BAV), higher training volumes do not correspond to a more rapid and significant deterioration of valvular function nor to an accelerated progression of aortic or cardiac chamber dimensions.

## Figures and Tables

**Table 1:** Anthropometric parameters modification between the first evaluation and the follow-up.

Parameters	First	Follow-Up	*p*-Value
Weight (kg)	69.6 ± 10.5	74.5 ± 10.4	<0.001
Height (cm)	177.0 ± 7.55	177.0 ± 8.3	0.794
BSA (m^2^)	1.85 ± 0.16	1.88 ± 0.15	0.233
BMI	22.6 ± 2.2	22.8 ± 2.3	0.573

**Table 2:** Differences between initial and follow-up assessment of key morpho-functional parameters in the bicuspid aortic valve (BAV) context in the two study groups (untrained and trained).

**Untrained at Follow-Up**			
**Parameters (Diameters or Velocities)**	**Mean ± SD** **(First Eval.)** **(mm or m/s)**	**Mean ± SD** **(Follow-Up)**	***p*-Value**
AOINS	22.71 ± 3.39	23.74 ± 2.65	0.217
AOVS	30.88 ± 6.6	33.4 ± 6.91	0.015
AOAN	21.9 ± 4.27	23.41 ± 3.34	0.041
AOSTJ	25 ± 4.26	26.12 ± 4.27	0.082
PAA	29.76 ± 4.91	32.16 ± 4.74	0.005
AA	22.8 ± 4.29	24.7 ± 4.21	0.004
TAOPV	1.71 ± 0.46	1.77 ± 0.42	0.416
DAOPV	1.57 ± 0.26	1.51 ± 0.27	0.351
**Trained at Follow-Up**			
**Parameters** **(Diameters or Velocities)**	**Mean ± SD** **(First Eval.)** **(mm or m/s)**	**Mean ± SD** **(Follow-Up)**	***p*-Value**
AOINS	22.4 ± 3	24.59 ± 5.1	0.065
AOVS	30.82 ± 4.75	33.1 ± 4.1	˂0.001
AOAN	22.9 ± 3	24.71 ± 4.11	0.023
AOSTJ	25.22 ± 3.67	26.58 ± 4.13	0.041
PAA	30.86 ± 5.78	32.23 ± 6.3	0.011
AA	23.25 ± 3.81	24.32 ± 4.32	0.033
TAOPV	1.5 ± 0.3	1.4 ± 0.21	0.016
DAOPV	1.62 ± 0.35	1.78 ± 3.96	0.035

AOINS: Aortic Insertion diameter; AOVS: Aortic Valve at Valsalva’s Sinus diameter; AOAN: Aortic Annulus diameter; AOSTJ: Sino-Tubular Junction diameter; PAA: Proximal Ascending Aorta diameter; AA: Aortic Arch diameter; TAOPV: Transaortic Peak Velocity; DAOPV: Descending Aorta Peak Velocity.

**Table 3:** Variation rate of the main echocardiographic parameters between first evaluation and follow-up, compared between the two groups (untrained and trained at follow-up).

Parameters	UFU–Variation Rate, % (Mean ± SD)	TFU–Variation Rate, % (Mean ± SD)	*p*-Value
AOINS	2.48 ± 12.46	7.75 ± 28.03	0.502
AOVS	1.76 ± 8.44	2.48 ± 8.31	0.776
AOAN	0.26 ± 11.19	4.75 ± 21.51	0.419
AOSTJ	2.14 ± 9.0	5.03 ± 12.93	0.466
PAA	5.02 ± 11.67	5.35 ± 11.22	0.926
AA	4.39 ± 10.36	2.01 ± 11.04	0.482
LVEDd	10.53 ± 5.27	20.88 ± 3.38	0.153
LVESd	2.08 ± 10.76	2.98 ± 14.0	0.199
IVSd	6.18 ± 18.5	1.32 ± 12.76	0.110
LVPWd	6.99 ± 15.3	1.93 ± 17.82	0.088
EF	−0.13 ± 8.68	6.12 ± 14.14	0.111
LAd	0.17 ± 14.01	6.45 ± 16.56	0.190
RVd	7.02 ± 16.43	−0.9 ± 14.65	0.133

## Data Availability

The raw data supporting the conclusions of this article will be made available by the authors on request.
